# Synchronous occurrence of hereditary gastric adenocarcinoma, gastrointestinal stromal tumor, and esophageal small cell and squamous carcinoma in situ: an extremely rare case report

**DOI:** 10.1186/s12885-017-3736-0

**Published:** 2017-11-07

**Authors:** Huijie Fan, Pei Lu, Li Xu, Yanru Qin, Jing Li

**Affiliations:** Department of Oncology, the First Affiliated Hospital of Zhengzhou University, No.1, Jianshe Road, Zhengzhou, 450000 China

**Keywords:** Synchronous tumors, Gastric adenocarcinoma, Gastrointestinal stromal tumors, Esophageal small cell carcinoma, Esophageal squamous carcinoma

## Abstract

**Background:**

Hereditary diffuse gastric carcinoma (HDGC) accounts for 1–3% of all gastric carcinomas. Gastrointestinal stromal tumors (GISTs) are the most common mesenchymal tumors in the gastrointestinal (GI) tract but they comprise fewer than 1% of all GI malignancies. Small-cell carcinoma (SmCC) is a rare histological type of esophageal carcinoma, accounting for 0.4% to 2.8% of all esophageal tumors. Co-occurrence of SmCC with esophageal tumors caused by squamous carcinoma is also very uncommon. Although multiple primary malignancies are no longer rare in clinical practice, the simultaneous appearance of HDGC, GIST, esophageal small cell and squamous carcinoma in situ is extremely rare and very few cases have been reported.

**Case presentation:**

We present a case of a 53 year-old woman with synchronous occurrence of four malignancies including HDGC, GIST, esophageal small cell- and local squamous carcinoma in situ. A total gastrectomy with D2 lymph node dissection and postoperative adjuvant chemotherapy with oxaliplatin and paclitaxel liposome were performed. After a 1-year follow-up, this patient was still in good condition with no evidence of recurrence.

**Conclusion:**

This is the unique case that describes the co-existence of the aforementioned four types of neoplasm. This case demonstrates that a diagnosis of gastric cancer does not preclude the presence of other malignancies and every case should be thoroughly analyzed to avoid missing other problems, which may worsen the prognosis.

## Background

Synchronous tumors are independent primary tumors occurring simultaneously [1]. Currently, more and more multiple primary malignant neoplasms have been found as a consequence of improvements in diagnostic methods and in the higher overall survival and life expectancy rate. However, synchronous occurrence of four malignancies is extremely rare and very few cases have been reported in English literature [2]. Here, we report a case of synchronous occurrence of HDGC, GIST, SmCC, and local esophageal squamous cell carcinoma (ESCC) in situ.

Gastric cancer (GC) is the third most common cause of cancer-related mortality worldwide [3]. There had been 246,660 new cases and 10,730 deaths in the United States alone during 2016 [4]. In China, gastric cancer is the second most common cancer and the third leading cause of cancer-related death as reported by the National Central Cancer Registry of China (NCCR) [5]. HDGC is an autosomal dominant cancer syndrome, accounting for 1–3% of all gastric carcinoma [6]. There are two established clinical diagnostic criteria for HDGC: (1) Confirmed diffuse gastric carcinoma (signet ring cell) are diagnosed in ≥2 first or second degree relatives, at least one patient <50 years of age, or (2) Documented diffuse gastric carcinoma are diagnosed in ≥3 first and second degree relatives, independent of age of onset [7]. The tumor suppressor gene CDH1 germline mutation (encoding E-cadherin) is deemed to be the most common molecular event in HDGC. However, this mutation is only detected in approximately 30–40% of cases, and in Asian countries, the proportion even smaller [8]. Recent publications have named other closely related mutations in gastric cancer with hereditary background: CTNNA1, BRCA2, ATM, MUC1, PSCA and PLCE1 [9].

## Case presentation

**Fig. 1:**
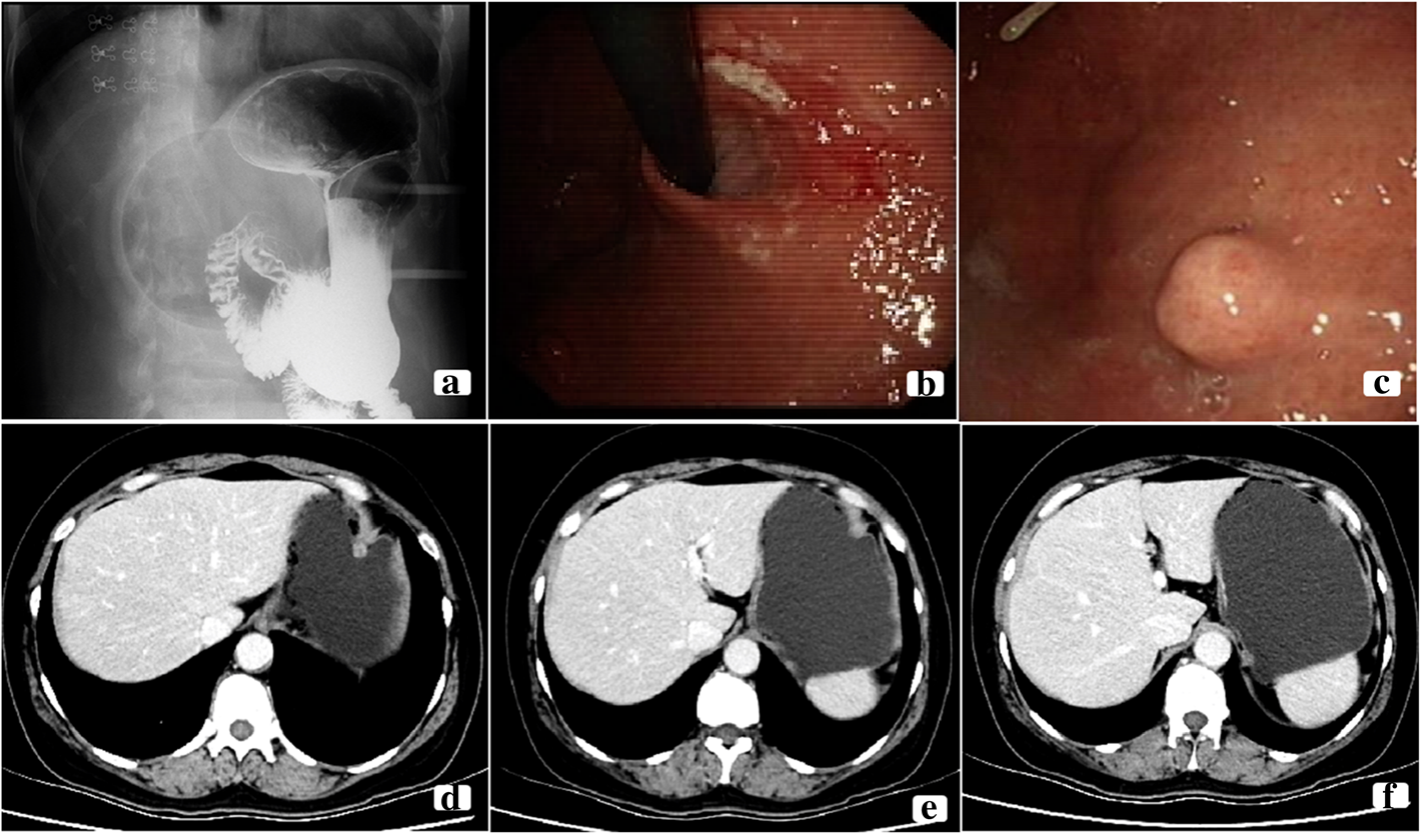
Imaging and endoscopic finds. **a** Barium swallow showing a small filling defect in the fundus of stomach. **b** Endoscopic examination showing a linear ulcer in the subcardiac region. **c** Endoscopic examination showing a polyp in the fundus of stomach. **d, e,** and **f** Enhanced abdominal CT scan showing obvious strengthening of the mucosal surface by thickening of the lateral wall of the lesser curvature of the fundus and body of stomach

A 53-year-old female who reported no discomfort underwent a medical examination and barium testing revealed a small filling-defect in the fundus of her stomach (Fig. 1a). Esophagogastroduodenoscopy(EGD) showed a linear ulcer in the subcardiac region and a polyp in the fundus of stomach (Fig. 1b and c). Biopsy revealed a high-grade intraepithelial neoplasia of glandular epithelium, with local intramucosal carcinoma. Her family history was significant: her mother had advanced gastric adenocarcinoma (deceased at age 50), her grandmother and an uncle had metastatic gastric carcinoma (diagnosed in their 50s, both deceased). She has no personal history of smoking or drinking alcohol. Physical examination was normal, showing good general condition with no obvious anemia or emaciation. Nothing abnormal was found in the cardiovascular or respiratory systems and there were no clinical symptoms within the abdomen. Blood chemistry and tumor markers were within normal limits. Metastatic work-up with a contrast-enhanced computed tomography (CT) scan of the chest and abdomen demonstrated the thickening lateral wall of the lesser curvature of the fundus and body of stomach. CT showed obvious strengthening of the mucosal surface of the thickening lateral wall without any lymph nodes or distant metastasis (Fig. 1d, e, and f).

A standard radical total gastrectomy with D2 lymph node dissection was performed on August 20, 2015. The histopathological examination revealed the following: a. well-differentiated gastric adenocarcinoma, invading the muscularis mucosa, with no carcinoma at the omentum; b. small cell carcinoma of esophagus, invading the submucosa, with local squamous cell carcinoma in situ; c. GIST with very low risk; d. no lymph node metastasis (0/18). The patient was discharged 3 weeks after the surgery and then underwent 6 courses of adjuvant chemotherapy consisting of oxaliplatin (130 mg/m^2^, intravenous) and paclitaxel liposome (75 mg/m^2^, intravenous).

## Pathological findings

### Microscopic findings

**Fig. 2:**
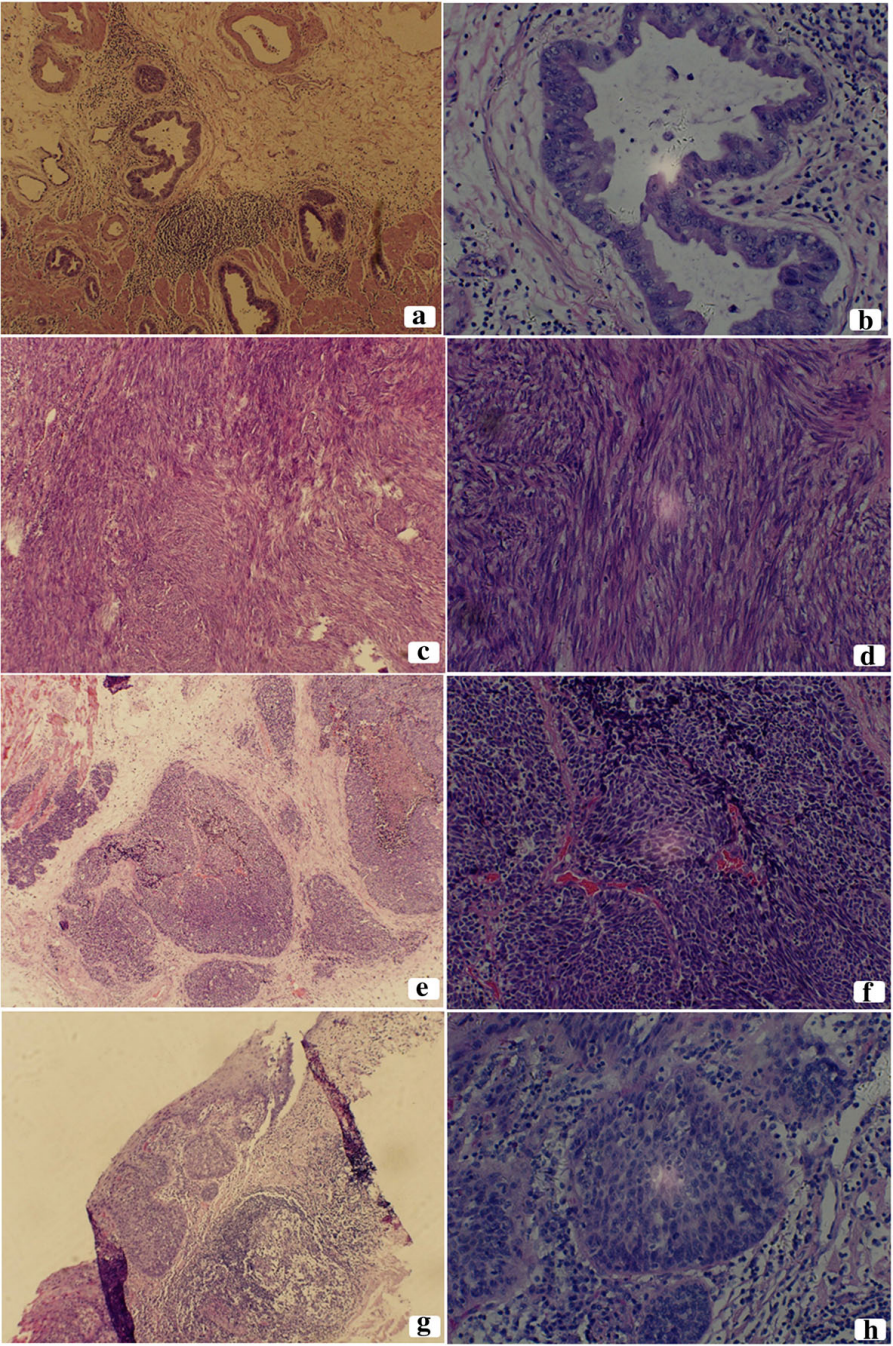
Photomicrograph showing the cytomorphological finds of the surgical resected specimen. **a** Gastric adenocarcinoma (H&E,×10) and **b** gastric adenocarcinoma (H&E,×40), (C) GIST (H&E,×10) and **d** GIST (H&E,×40), **e** esophageal small cell carcinoma (H&E,×10) and **f** esophageal small cell carcinoma (H&E,×40), **g** esophageal squamous cell carcinoma (H&E,×10) and **h** esophageal squamous cell carcinoma (H&E,×40)

The gastric cancer cells presented adenocarcinoma features, which were in adenoid arrangement with obvious atypia and karyokinesis (Fig. 2a and b). The submucosal gray mass of the stomach consisted of spindle cell proliferation with uniform tapering nuclei and indistinct syncytial cytoplasm (Fig. 2c and d). The esophageal lesions were characterized by small, round, or ovoid shape, scarce cytoplasm, and an inconspicuous or absent nucleolus (Fig. 2e and f). In local mucosa of the esophagus showed heterogenic squamous epithelium spread the whole epithelial cell stroma, with no breakage of the basement membrane (Fig. 2g and h). The distance of proximal resection margin is 3 cm up from the cardiac area of the stomach, and the distal resection margin reached the duodenal bulb.

### Immunohistochemistry

**Fig. 3:**
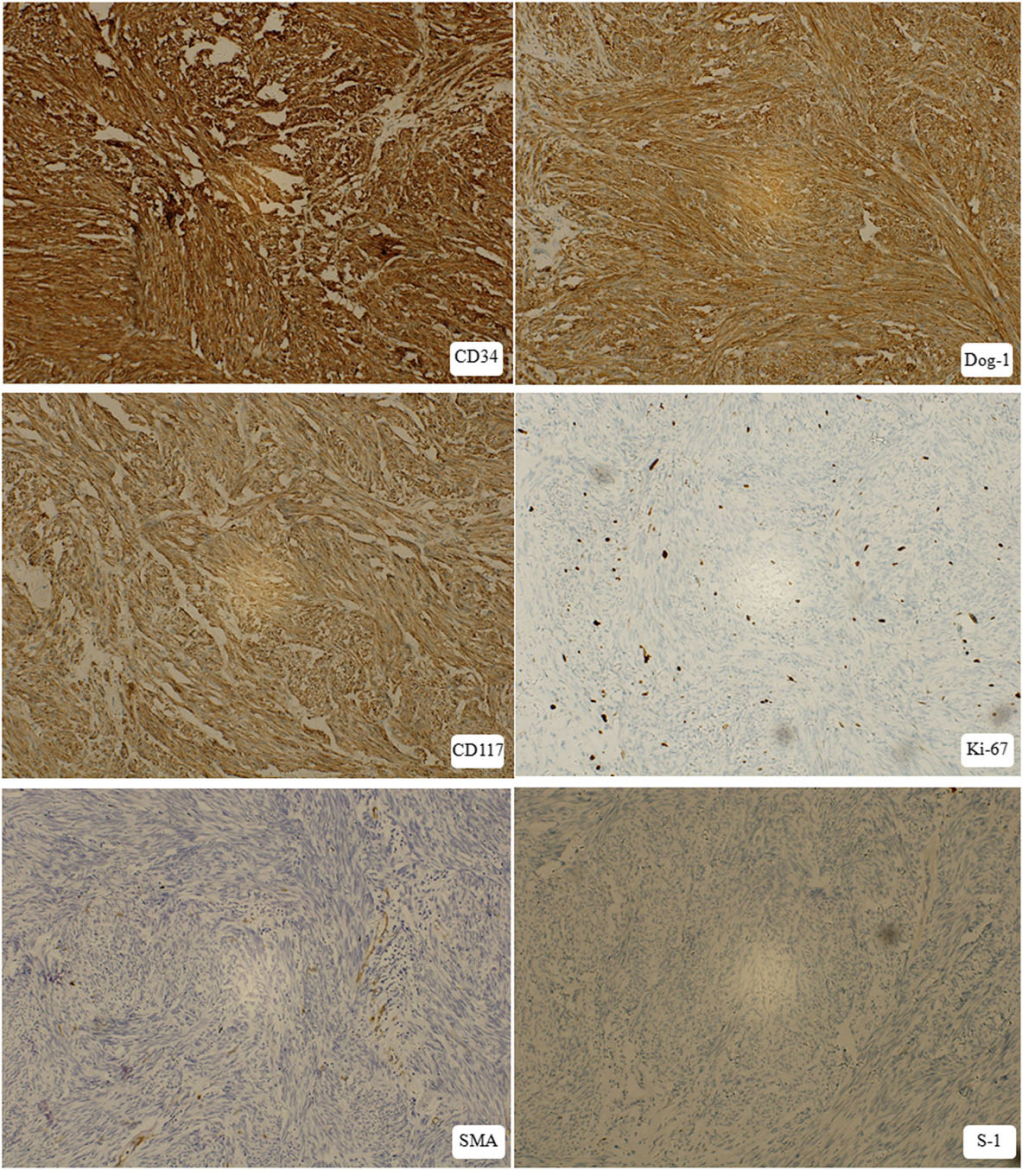
Photomicrograph showing immunohistochemical stains of GIST (IHC × 10). Positive for CD34, Dog-1, Ki-67(2%), CD117, and negative for Desmin, SMA and S-1

The cells of GIST were stained positive for CD34, Dog-1, Ki-67 (2%), CD117, and negative for Desmin, SMA and S-1 (Fig. 3).

**Fig. 4:**
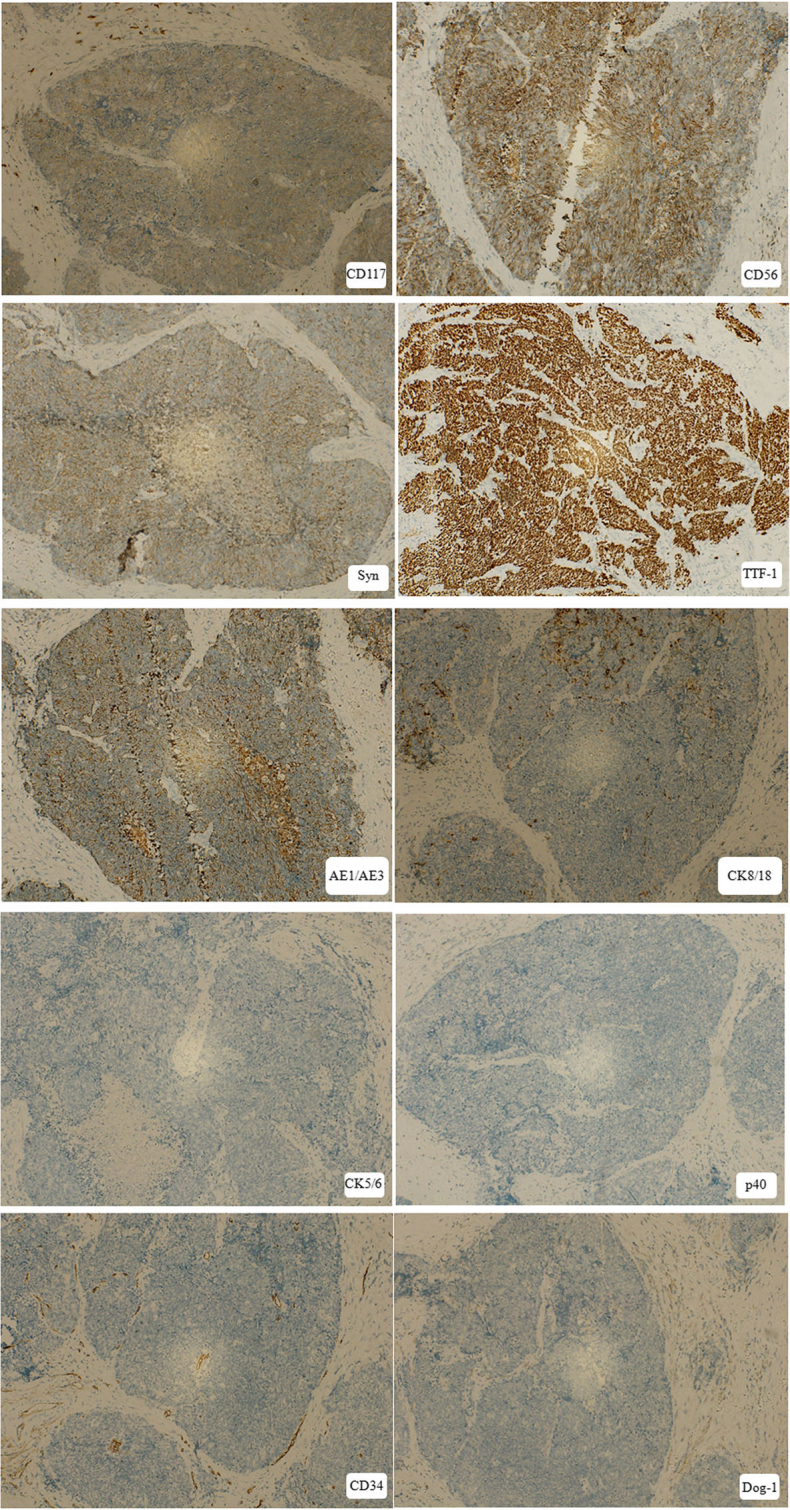
Photomicrograph showing immunohistochemical stains of SmCC (IHC × 10). Positive for CK8/18(focal+), CD (56), Syn, TTF-1 and AE1/AE3, and negative for CK5/6, p40, CD34, Dog-1

The cells of SmCC were stained positive for CK8/18 (focal+), CD (56), Syn, TTF-1 and AE1/AE3, and negative for CK5/6, p40, CD34, Dog-1 (Fig. 4).

On the basis of the microscopic and immunohistochemical findings, the diagnosis was gastric adenocarcinoma with synchronous gastrointestinal stromal tumor (GIST) (very low-risk), esophageal small cell carcinoma, and local squamous cell carcinoma in situ.

### Follow-up

The patient is still asymptomatic as of the writing of this report, and the results of physical examinations remained normal. Except for the presence of granuloctopenia, laboratory data were all normal at the time of discharge after her six courses of treatment (Grade 1), but there were no significant complications. One year after the start of treatment, the patient was still in good general condition and the disease was stable.

## Discussion

Multiple primary cancers are frequent findings in recent years, but triple or quadruple cancers occur in less than 0.5%. Among these patients, one-third is gastric cancers. It was reported that the most common sites for synchronous gastric cancer were head and neck, esophagus, lung and kidney. Here, we reported an extremely rare case of synchronous occurrence of four malignancies, including HDGC, GIST, and esophageal small cell and local squamous carcinoma in situ. To our knowledge, this is the first case of four primary tumors occurring simultaneously in a single person’s digestive tract reported in English literature.

Although this patient was the only family member diagnosed and treated at our facility (all others were deceased), her strong family history suggests that genetic testing could give her family the information they need to take potentially life-saving measures. Consequently, SNPs rs2294008 C > T in prostate stem cell antigen (PSCA) gene were found in the current study using next-generation sequencing (NGS). This gene, which has been reported to be involved in the regulation of gastric epithelial-cell proliferation and significantly closely associated with increased risk of diffuse-type gastric cancer (DGC) with hereditary background [10]. However, no important deleterious mutations were observed in other genes identified as closely related to HDGC, such as CDH1, ATM, BRCA2, CTNNA1, MSR1, PALB2, PRSS1, SDHB, or STK11.

Very little of the mechanisms underlying the carcinogenesis of gastric cancer are fully understood. Infectious agents (i.e., *Helicobacter pylori* (HP), Epstein-Barr virus (EBV)), behavioral factors (i.e., alcohol consumption, cigarette smoking, nitrated foods), and genetic background were associated with the risk of developing gastric cancer. Human PSCA gene maps on chromosome 8q24.2 and encodes an 123 amino acid cell surface protein which is a member of the Ly-6/Thy-1 family having an important function on cell adhesion, proliferation, and survival [11]. rs2294008 C > T polymorphism is the most extensively studied SNP in this gene and it has been shown to be significantly closely associated with increased overall cancer risk, especially for gastric cancer [12]. The mechanism and physiological function are not fully understood, in vitro experiments have demonstrated that the PSCA rs2294008T might decrease the transcriptional activity of the host gene by recruiting transcription factor Yin Yang 1 (YY1) to its promoter and eventually predispose gastric epithelial cells to GC development [13]. In addition to PSCA, the CDH1 gene is deemed to be the most common mutation in HDGC. Unfortunately, this germline mutation was not found in this case. Considering the strong family history, relatives of this patient are strongly advised to undergo genetic testing and screening endoscopic gastric biopsy evaluations.

GIST is a rare cancer but still the most common type of mesenchymal tumor in the GI tract [12]. The tumor originates from Cajal cells or interstitial pacemaker cells [13, 14]. As in this case, small GISTs are usually detected incidentally during surgery to address other diseases, and the majority show a low level of mitotic activity [15]. In previous studies, less than 20% of synchronous GISTs and other primary tumors are coincidentally diagnosed, and more than a half of these patients presented lesions in stomach [16, 17]. It has been reported that among the cancers occurring alongside non-GIST cancer, gastric adenocarcinoma accounted for 42.6%, and ESCC for 50% [15].

ESCC accounts for 90% of cases among Asian countries, while SmCC is a rare histological type of esophageal carcinoma, accounting for 0.4% to 2.8% of all esophageal carcinoma [18, 19]. Actually, the synchronous occurrence of squamous carcinoma and SmCC is also extremely uncommon. SmCC is characterized as a highly aggressive malignant by early dissemination and poor prognosis, with median survival ranging from 3.1 to 15.5 months [20–22]. The standard treatment protocol for such disease has not been established, although surgical resection, radiotherapy, and multidrug chemotherapy have been used either alone or in combination [23, 24]. At present, there are two established hypotheses regarding the histological origin of SmCC: (1) SmCC originates from the amine precursor uptake and decarboxylase (SPUD) cells of the submucosal gland or stratum basal and (2) SmCC originates from pluripotent stem cells of the endoderm [25, 26]. Because most of the stem cells may be differentiated into squamous cell carcinoma and some did differentiate into small cell carcinoma, this may be the histological basis of the coexistence of SmCC and ESCC.

This is the first documented case of the simultaneous appearance of four primary malignancies in any esophagogastric location reported in English. No convincing explanation is still given for this coexistence. Simple coincidence could be the most reasonable explanation.

## Conclusion

In summary, this report describes an extremely rare case of synchronous occurrence of HDGC, GIST, and esophageal small cell and squamous carcinoma in situ. The nature of the association between them is unknown, and further research is needed to explain the simultaneous tumors, if there is such. This case demonstrates that the diagnosis of one type of cancer should not exclude the presence of other malignancies and every case should be thoroughly analyzed to avoid overlooking any relevant condition, which may worsen the prognosis.

## Abbreviations

CT: Computed tomography
DGC: Diffuse-type gastric cancer
EBV: Epstein- Barr virus
ESCC: Esophageal squamous cell carcinoma
GC: Gastric cancer
GIST: Gastrointestinal stromal tumor
GPI: Glycosylphosphatidyl-inositol
HDGC: Hereditary diffuse gastric cancer
HP: Helicobacter pylori
NCCR: National Central Cancer Registry of China
NGS: Next-generation sequencing
PSCA: Prostate stem cell antigen gene
SmCC: Small cell carcinoma
SNP: Single nucleotide polymorphism
YY1: Yin Yang 1
